# *Pseudofornicia* gen. n. (Hymenoptera, Braconidae, Microgastrinae), a new Indo-Australian genus and one new species from Vietnam

**DOI:** 10.3897/zookeys.524.6158

**Published:** 2015-09-30

**Authors:** Cornelis van Achterberg, Khuat Dang Long, Xue-xin Chen, Lan-shao You

**Affiliations:** 1 Department of Terrestrial Zoology, NCB Naturalis, Postbus 9517, 2300 RA Leiden, The Netherlands / Key Laboratory of Resource Biology and Biotechnology in Western China (Northwest University), Ministry of Education; School of Life Sciences, Northwest University, 229 North Taibai Road, Xi’an, Shaanxi 710069, China; 2 Institute of Ecology & Biological Resources, Vietnam Academy of Science & Technology, 18 Hoang Quoc Viet Road, Cau Giay, Ha Noi, Vietnam; 3 Institute of Insect Sciences, Zhejiang University, Zijingang Campus, Yuhangtang Road 866, Hangzhou 310058, China; 4 College of Bio-Safety Science and Technology, Hunan Agriculture University, Changsha 410128, China

**Keywords:** *Fornicia*, *Diolcogaster*, *Buluka*, key, new genus, Oriental, China, Australia

## Abstract

*Pseudofornicia* **gen. n.** (Hymenoptera: Braconidae: Microgastrinae) is described (type species: *Pseudofornicia nigrisoma* **sp. n.** from Vietnam) including three Oriental (type species, *Pseudofornicia flavoabdominis* (He & Chen, 1994), **comb. n.** and *Pseudofornicia vanachterbergi* Long, (**nom. n.** for *Fornicia achterbergi* Long, 2007; not *Fornicia achterbergi* Yang & Chen, 2006) and one Australian species (*Pseudofornicia commoni* (Austin & Dangerfield, 1992), **comb. n.**). Keys to genera with similar metasomal carapace and to species of the new genus are provided. The new genus shares the curved inner middle tibial spur, the comparatively small head, the median carina of the first metasomal tergite and the metasomal carapace with *Fornicia* Brullé, 1846, but has the first tergite movably joined to the second tergite and the third tergite 1.1–1.6 × as long as the second tergite medially and is flattened in lateral view. One of the included species is a primary homonym and is renamed in this paper.

## Introduction

During the review of the genus *Fornicia* Brullé, 1846 (Braconidae: Microgastrinae) by the first two authors (van Achterberg and Long, in prep.), it was discovered that some of its Indo-Australian species and a new species from Vietnam did not fit in *Fornicia* because the first tergite of the carapace is movably connected to the second tergite. During the evolution of the Microgastrinae a carapace was independently developed several times in various ways (Mason 1981), but the exact phylogenetic history of this character and its states is still largely unknown. A new genus (*Pseudofornicia* gen. n.) is named herein to accommodate for these very similar but overall smaller species.

## Material and methods

For identification of the subfamily Microgastrinae, see van Achterberg (1990, 1993), for identification of the genus *Fornicia*, see Mason (1981), for references to the genus *Fornicia* and other genera mentioned in this paper, see Yu et al. (2012). Photographic images were made with the Keyence VHX-5000 digital microscope and processed with Adobe Photoshop CS5, mostly to adjust the size and background. Morphological terminology follows van Achterberg (1988, 1993), including the abbreviations for the wing venation. Measurements are taken as indicated by van Achterberg (1988) for the length and the width of a body part the maximum length and width is taken, unless otherwise indicated. The length of the mesosoma is measured from the anterior border of the mesoscutum till the apex of the propodeum and of the first tergite from the posterior border of the adductor till the medio-posterior margin of the tergite.

The specimens are deposited in the following collections: Institute of Insect Sciences, Zhejiang University (ZJUH), Hangzhou; Institute of Ecology & Biological Resources (IEBR), Hanoi, Vietnam National Museum of Nature (VNMN), Hanoi, Naturalis Biodiversity Center (RMNH), Leiden and Australian National Insect Collection (ANIC), Canberra. In the keys we use in some couplets “if”, “then” or “and” in bold to be explicit that in those cases more than one character state has to be considered. Additional non-exclusive characters are between brackets.

### Key to microgastrine genera with complete metasomal carapace

(only to females of genera with carapace covering most of metasoma and having the dorsal face of the first tergite shorter than the second tergite)

**Table:** 

1	Three anterior metasomal tergites forming a strongly convex carapace in lateral view, with first tergite immovably joined to second tergite **and** prepectal carina present behind fore coxae; outer aspect of scapus strongly concave apically; axilla of scutellum wide laterally, lamelliform and sub-vertically curved up above base of hind wing; head unusually small, 0.7–0.8 × as wide as mesoscutum in dorsal view; [vein r-m of fore wing absent; vein 1-SR of fore wing linear with vein 1-M; vein cu-a of hind wing mostly sinuate and inclivous]	***Fornicia* Brullé, 1846**
–	Three anterior tergites forming a flattened carapace in lateral view and first tergite movably joined to second tergite (best seen laterally as a distinct separation between both tergites: Figs 4, 14, 24), **if** immovably joined (some *Diolcogaster* and *Xanthapanteles*) **then** prepectal carina completely absent; outer aspect of scapus often truncate apically or nearly so, but with oblique apex in *Pseudofornicia* (Fig. 10); axilla of scutellum narrow laterally, less lamelliform and almost flat above base of hind wing, rarely rather curved up (e.g. *Buluka*); head medium-sized, 0.8–1.0 × as wide as mesoscutum in dorsal view	**2**
2	Vein r-m of fore wing absent (Figs 2, 22); second suture of metasoma curved and together with lateral grooves of medial area forming a more or less X-shaped figure (Figs 3, 13, 23); vein 1-SR of fore wing 0.3–0.4 × as long as vein 1-M (Figs 2, 12); dorsal carinae of first metasomal tergite united into median carina posteriorly and with a lamella separating dorsal and anterior face of tergite (Figs 13, 23); medio-longitudinal carina of propodeum absent (Fig. 13); height of head 0.5–0.7 × height of mesosoma (Figs 1, 11); third tergite 1.1–1.6 × as long as second tergite medially (Figs 2, 13, 23)	***Pseudofornicia* van Achterberg, gen. n.**
–	Vein r-m of fore wing present; second suture straight and without X-shaped impression; vein 1-SR of fore wing 0.1–0.3 × as long as vein 1-M; dorsal carinae of first metasomal tergite separated throughout and without a lamella separating dorsal and anterior face of tergite; propodeum with complete medio-longitudinal carina; height of head 0.8–0.9 × height of mesosoma; third tergite 1.0–2.0 × as long as second tergite medially	**3**
3	Second tergite with distinct medial area surrounded by grooves and tergite about as long as third tergite; second submarginal cell of fore wing (“areolet”) petiolate and hardly wider than width of surroundings veins; fourth and following tergites of ♀ more or less exposed	***Diolcogaster* Ashmead, 1900**, p.p.
–	Second tergite without distinct medial area, at most vaguely indicated and tergite about half as long as third tergite; second submarginal cell of fore wing sessile and distinctly wider than width of surroundings veins; fourth and following tergites of ♀ retracted	***Buluka* de Saeger, 1948**

## Systematics

### Pseudofornicia

Taxon classificationAnimaliaHymenopteraBraconidae

van Achterberg gen. n.

http://zoobank.org/60B6A212-2344-493B-9168-F4E277EA8977

Figs 1 , 2–10 , 11 , 12–20 , 21 , 22–30

#### Etymology.

The specific name is derived from “pseudos” (Greek for “fallacy”) and the generic name *Fornicia* Brullé, because it is similar to that genus. Gender: feminine.

#### Type species.

*Pseudofornicia nigrisoma* van Achterberg & Long, sp. n.

#### Diagnosis.

**Figure 1.**
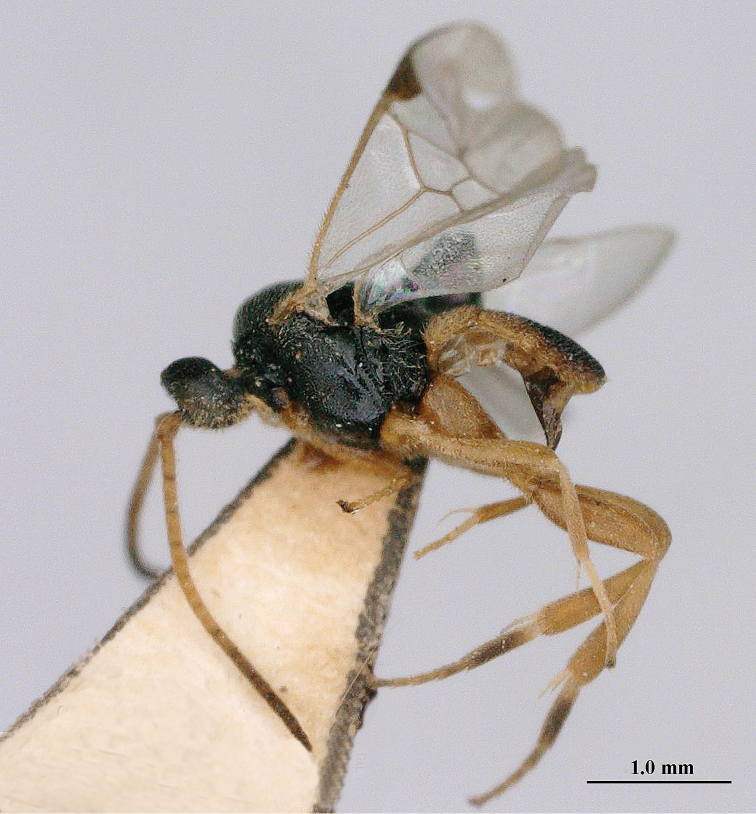
*Pseudofornicia flavoabdominis* He & Chen, female, paratype, habitus lateral.

**Figures 2–10.**
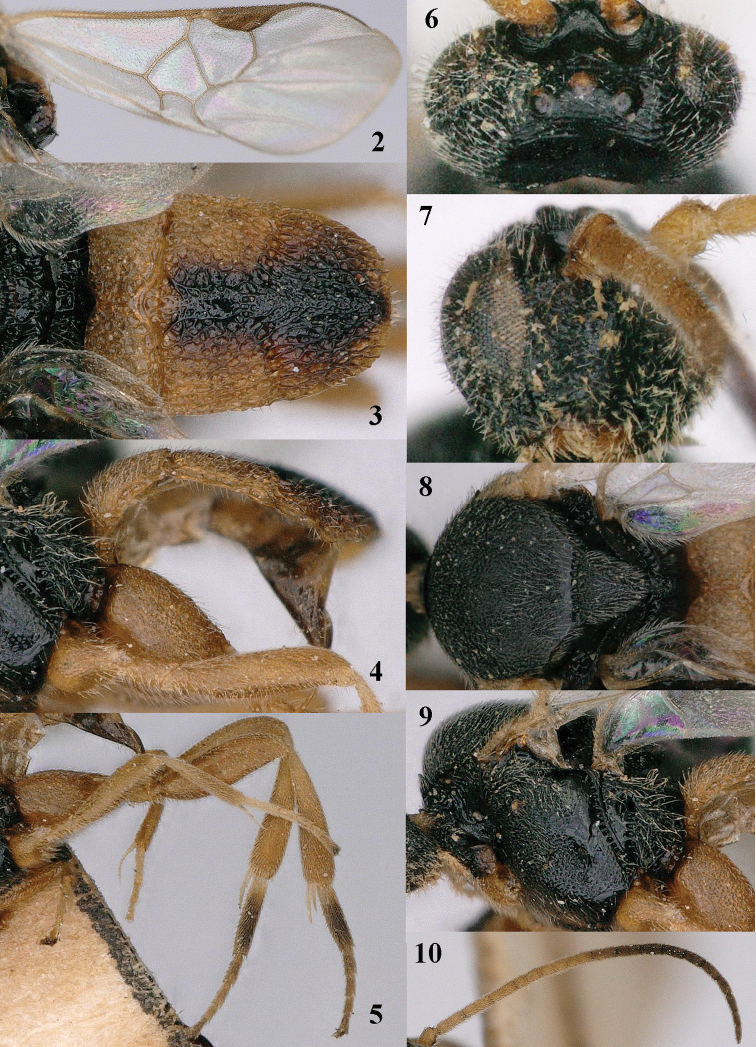
*Pseudofornicia flavoabdominis* He & Chen, female, paratype. **2** fore wing **3** metasoma dorsal **4** metasoma lateral **5** hind leg **6** head dorsal **7** head anterior **8** mesosoma dorsal **9** mesosoma lateral **10** antenna.

**Figure 11.**
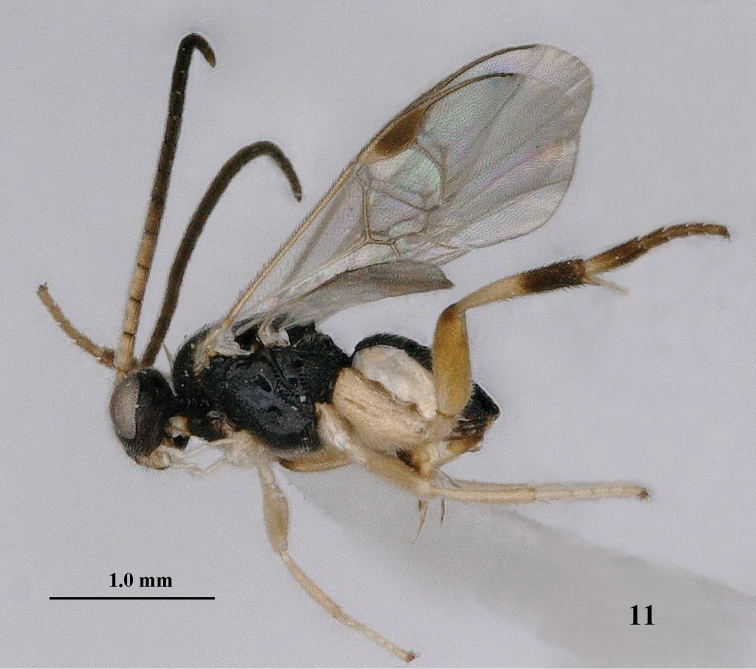
*Pseudofornicia nigrisoma* sp. n., female, holotype, habitus lateral.

**Figures 12–20.**
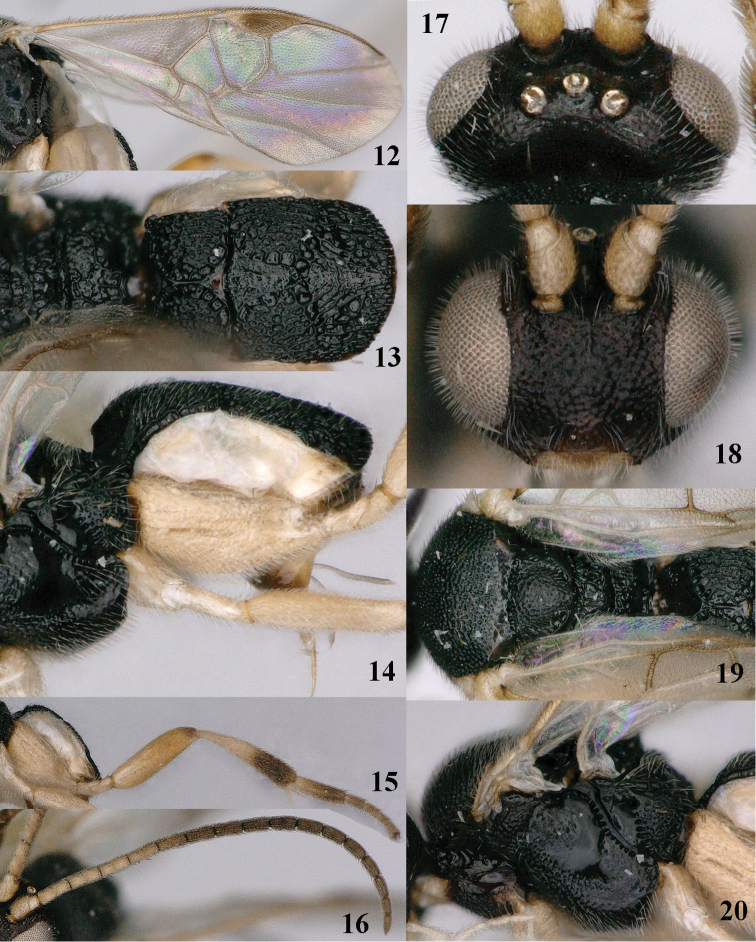
*Pseudofornicia nigrisoma* sp. n., female, holotype. **12** fore wing **13** metasoma dorsal **14** metasoma lateral **15** hind leg **16** antenna **17** head dorsal **18** head anterior **19** mesosoma dorsal **20** mesosoma lateral.

Height of head 0.5–0.7 × height of mesosoma in lateral view (Figs 1, 11) and width of head 0.8–0.9 × width of mesoscutum; scapus moderately oblique apically; prepectal carina absent; axilla curved up over base of hind wing; metanotum with lobe-shaped protuberance postero-dorsally; medio-longitudinal carina of propodeum absent (Fig. 13); vein r-m of fore wing absent (Figs 2, 22); vein 1-SR of fore wing 0.3–0.4 × as long as vein 1-M (Figs 2, 12); inner middle tibial spur as long as basitarsus and curved (Fig. 1); three anterior tergites of metasoma forming a flattened carapace covering most of metasoma dorsally (Figs 4, 14, 24); first tergite movably joined to second tergite (Fig. 4); dorsal carinae of first metasomal tergite united into median carina posteriorly and with a lamella separating dorsal and anterior face of tergite (Figs 13, 23), anterior face smooth and flat; second suture of metasoma sinuate, crenulate and together with lateral grooves of medial area forming a more or less X-shaped figure (Figs 3, 13, 23); third tergite 1.1–1.6 × as long as second tergite medially (Figs 2, 13, 23); fourth-sixth tergites more or less sclerotized; ovipositor short and decurved; ovipositor sheath largely glabrous, narrow and only apically with some long setae; hypopygium of female fully sclerotized and acute apically. Males unknown.

**Figure 21.**
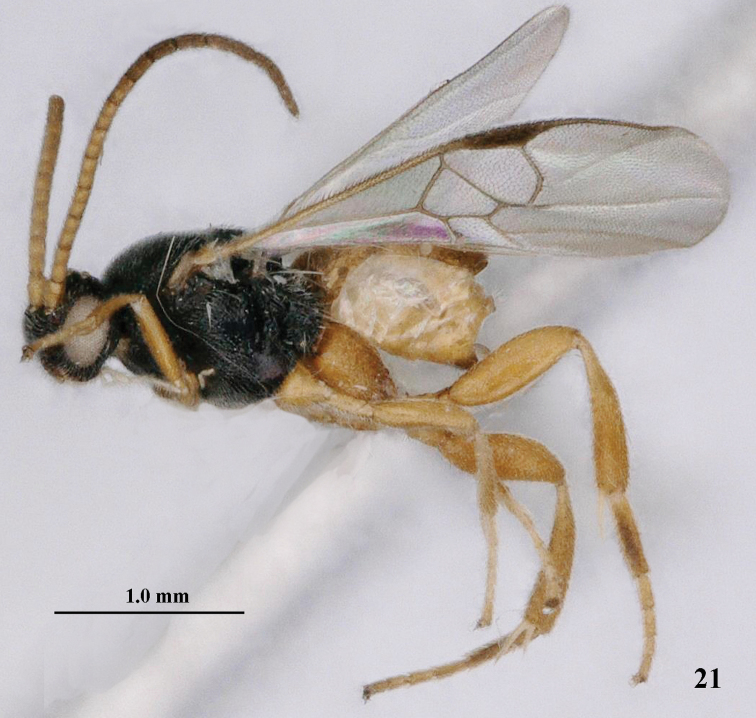
*Pseudofornicia vanachterbergi* nom. n., female, holotype, habitus lateral.

**Figures 22–30.**
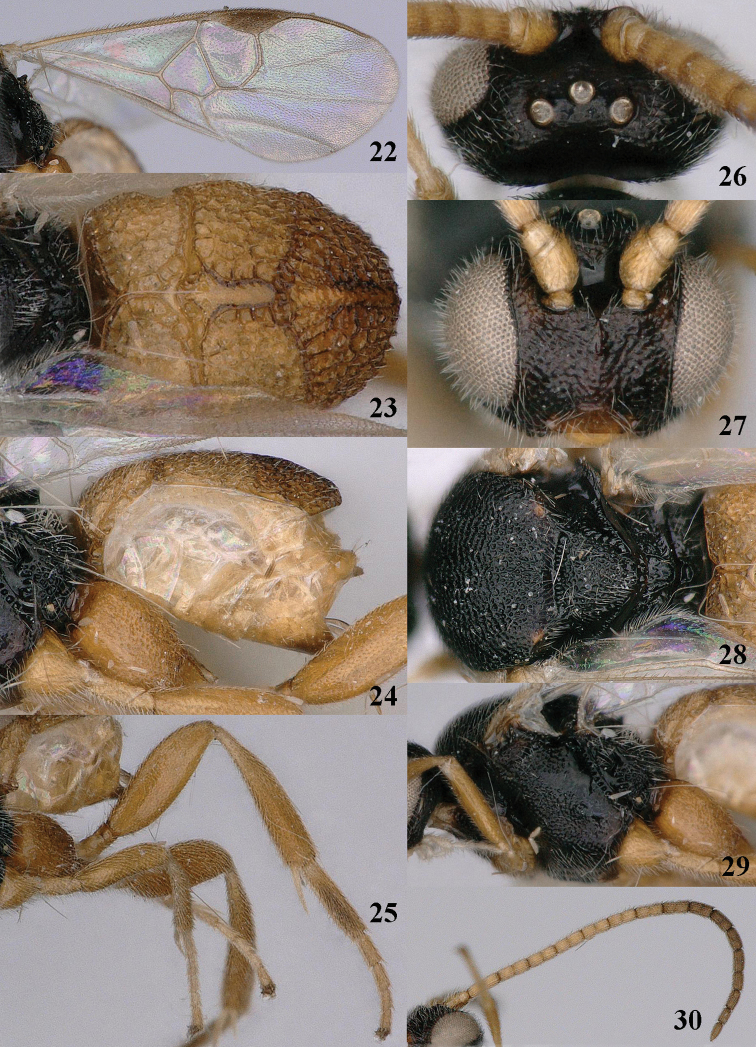
*Pseudofornicia vanachterbergi* nom. n., female, holotype. **22** fore wing **23** metasoma dorsal **24** metasoma lateral **25** hind leg **26** head dorsal **27** head anterior **28** mesosoma dorsal **29** mesosoma lateral **30** antenna.

#### Distribution.

Indo-Australian.

#### Biology.

Unknown, but the species of the very similar genus *Fornicia* are koinobiont endoparasitoids of limacodid caterpillars (Yu et al. 2012).

#### Comments.

The genus will run in the key to world genera of Microgastrinae by Mason (1981) to the genus *Fornicia* Brullé. The new genus can be separated as follows:

**Table:** 

1	Third tergite 1.1–1.6 × as long as second tergite medially and flattened in lateral view; first tergite movably joined to second tergite; second tergite with wide and anteriorly widened medial area; second suture of metasoma curved and together with lateral grooves of medial area more or less X-shaped; head 0.8–1.0 × as wide as mesoscutum; prepectal carina absent; fourth-sixth tergites more or less sclerotized; scapus moderately oblique apically	***Pseudofornicia* van Achterberg, gen. n.**
–	Third tergite 0.5–0.9 × as long as second tergite medially and curved in lateral view; first tergite immovably joined to second tergite; second tergite with narrow and anteriorly parallel-sided medial area; second suture of metasoma straight and not connected to lateral grooves of medial area and not X-shaped; head 0.7–0.8 × as wide as mesoscutum; prepectal carina present behind fore coxa; fourth-sixth tergites mainly membranous; scapus strongly oblique apically	***Fornicia* Brullé**

#### Key to species of the genus *Pseudofornicia*

**Table:** 

1	Medial area of second metasomal tergite wide triangular (Fig. 46 in Austin and Dangerfield 1992); fore wing with two dark patches; scutellum with a slender (in lateral view tooth-like, but in dorsal view obtuse) protuberance posteriorly; height of head 0.5 × height of mesosoma in lateral view; median carina of first tergite 0.3–0.4 × as long as dorsal face of tergite; [metasoma black]; Australian region (Australia: Queensland)	***Pseudofornicia commoni* (Austin & Dangerfield, 1992), comb. n.**
-	Medial area of second tergite largely subparallel-sided and only anteriorly widened (Figs 3, 23) or vase-shaped (Fig. 13); fore wing without dark patches; scutellum without protuberance, at most with a more or less up curved subposterior rim; height of head 0.6–0.7 × height of mesosoma in lateral view (Figs 1, 11); median carina of first tergite nearly as long as dorsal face of tergite (Figs 3, 13, 23); Oriental region	**2**
2	Metasoma black dorsally and parallel-sided (Fig. 13); vein m-cu of fore wing about as long as vein 2-SR+M (Fig. 12); apical half of hind tibia dark brown (Figs 11, 15); height of head 0.7 × height of mesosoma in lateral view (Fig. 11); propodeum without elevated medio-basal area (Fig. 13); medial area of second tergite vase-shaped (Fig. 13); median length of third tergite 1.2 × second tergite (Fig. 13); vein cu-a of hind wing nearly straight (Fig. 19)	***Pseudofornicia nigrisoma* van Achterberg & Long, sp. n.**
–	Metasoma brownish-yellow dorsally, at most second and third tergites medially dark brown and roundly narrowed posteriorly (Figs 3, 23); vein m-cu of fore wing shorter than vein 2-SR+M (Figs 2, 22); apical half of hind tibia yellowish brown (Figs 5, 25); height of head 0.6 × height of mesosoma in lateral view (Fig. 1); propodeum with small elevated medio-basal area (Fig. 3); medial area of second tergite largely subparallel-sided and only anteriorly widened (Figs 3, 23); median length of third tergite 1.3–1.5 × second tergite (Figs 3, 23); vein cu-a of hind wing moderately sinuate (Figs 8, 24)	**3**
3	Head 0.8 × as wide as mesoscutum; anterior half of medial area of second metasomal tergite largely sculptured (especially laterally) and more gradually narrowed posteriorly (Fig. 3); first tergite near median carina hardly depressed and X-shaped groove superficial (Fig. 3, but posteriorly impressed); first discal cell nearly as setose as apical third of fore wing; apical rim of scutellum remaining far below upper level of scutellum; second and third tergites dark brown medially (Fig. 3); third tergite densely finely reticulate medially (Fig. 3)	***Pseudofornicia flavoabdominis* (He & Chen, 1994) comb. n.**
-	Head 0.9 × as wide as mesoscutum; anterior half of medial area of second metasomal tergite largely smooth, except some punctures laterally and more abruptly narrowed posteriorly (Fig. 23); first tergite near median carina depressed because of distinctly impressed X-shaped groove (Fig. 23); first discal cell less setose than apical third of fore wing; apical rim of scutellum nearly reaching upper level of scutellum (Fig. 29); second and third tergites brownish yellow medially (Fig. 23); third tergite coarser reticulate medially (Fig. 23)	***Pseudofornicia vanachterbergi* Long, nom. n.**

### Pseudofornicia commoni

Taxon classificationAnimaliaHymenopteraBraconidae

(Austin & Dangerfield, 1992) comb. n.

Fornicia commoni Austin & Dangerfield, 1992: 29–31, figs 44–47 (only holotype (ANIC) known: Australia, Queensland, 25 miles N of Gin Gin; not examined).

#### Diagnosis.

Easily to recognize by having the second metasomal tergite with a large triangular medio-basal area (Fig. 46 in Austin and Dangerfield 1992), the fore wing with two dark patches, the scutellum with a slender (in lateral view tooth-like, but in dorsal view obtuse) protuberance and the median carina of the first tergite short.

#### Distribution.

Australia (Queensland).

#### Biology.

Unknown. Holotype collected in March.

### Pseudofornicia flavoabdominis

Taxon classificationAnimaliaHymenopteraBraconidae

(He & Chen, 1994) comb. n.

Figs 1 , 2–10

Fornicia flavoabdominis He & Chen (in Chen et al.), 1994: 130–131, 134, figs 22–26.

#### Type material.

Holotype ♀ (ZJUH), “[S. China], Zhejiang, Linan Xian, Yuqian, 2.vi.1958, Hu Cui”, “5845.1”. Paratype (ZJUH): 1 ♀, same label data, but “5845.1P”.

#### Additional material.

1 ♀ (ZJUH) from Zhejiang, viii.1984.

#### Diagnosis.

Metasoma brownish yellow, at most second and third tergites medially dark brown; first tergite moderately coarsely reticulate (Fig. 3); medial area of second metasomal tergite gradually narrowed, largely sculptured and posteriorly narrower than medial area of third tergite anteriorly (Fig. 3); second tergite 0.6–0.7 × as long as third tergite and third tergite with moderately wide parallel-sided elevation and densely finely reticulate medially; scutellum in lateral view not protruding apically, with narrow curved lamella remaining far below upper level of scutellum, medially punctate or distinctly rugose; hind leg (except largely dark brown basitarsus) brownish-yellow.

#### Distribution.

China (Zhejiang).

#### Biology.

Unknown. Adults collected in June and August.

### Pseudofornicia nigrisoma

Taxon classificationAnimaliaHymenopteraBraconidae

van Achterberg & Long sp. n.

http://zoobank.org/9F821EAE-CC41-4370-92C4-90A15C01D87B

Figs 11 , 12–20

#### Type material.

Holotype, ♀ (IEBR), “Vietnam: Ha Tinh, Huong Son, 18°22'N, 106°13'E, 300 m, 20.iv.–1.v.1998, Malaise [trap], AMNH, K. Long”. Paratypes: 1 ♂ (VNMN), same data, except 2–11.v.1998, Mic.739; 1 ♀ (RMNH), same data, except 900 m, 5.v.1998, Mic. 1049.

#### Diagnosis.

Height of head 0.7 × height of mesosoma in lateral view (Fig. 11) and its width equal to width of mesoscutum; vein m-cu of fore wing about as long as vein 2-SR+M (Fig. 12); fore wing without dark patches; vein cu-a of hind wing nearly straight (Fig. 19); scutellum punctate, without protuberance, with a more or less up curved subposterior rim remaining far below upper level of scutellum; propodeum without elevated medio-basal area (Fig. 13); apical half of hind tibia dark brown (Figs 11, 15); metasoma black dorsally and parallel-sided (Fig. 13); median carina of first tergite nearly as long as dorsal face of tergite (Fig. 13); medial area of second tergite vase-shaped (Fig. 13); median length of third tergite 1.2 × second tergite (Fig. 13); length of body 2.4–2.5 mm.

#### Description.

Holotype, ♀, length of body 2.4 mm, of fore wing 2.7 mm.

*Head.* Height of head 0.7 × height of mesosoma in lateral view (Fig. 11) and its width equal to width of mesoscutum; antennal articles 18, length of third article 1.1 × fourth segment, length of third, fourth and penultimate segments 3.3, 3.0 and 2.2 × their width, respectively (Fig. 16); maxillary palp 0.9 × height of head; malar space 0.7 × as long as basal width of mandible; length of eye in dorsal view 2.2 × temple; temple directly narrowed posteriorly (Fig. 17); POL:OD:OOL= 12:5:5; face pimply with smooth interspaces; frons shiny and smooth, vertex laterally and temple with superficial rugae (Fig. 17).

*Mesosoma*. Length of mesosoma 1.3 × its height; propleuron densely rugose; pronotum shiny, with some rugae and smooth posteriorly; mesopleuron densely rugose-punctate anteriorly and remainder largely smooth (Fig. 20); mesosternum shiny and moderately densely punctate; mesoscutum with satin sheen, densely punctate and notauli indicated by reticulate-punctate bands; scutellum rather convex, punctate, without protuberance, its subposterior rim slightly up curved and remaining far below upper level of scutellum; propodeum areolate and rather shiny, without elevated medio-basal area (only with small areola) or median carina (Fig. 13).

*Wings*. Fore wing: vein m-cu about as long as vein 2-SR+M (Fig. 12); vein 1-SR 0.35 × as long as vein 1-M; vein 1-R1 1.2 × as long as pterostigma; r:2-SR:2-SR+M = 10:10:7; vein 1-CU1 half as long as vein 2-CU1. Hind wing: vein cu-a nearly straight and its surroundings glabrous; vein M+CU about as long as vein 1-M.

*Legs*. Hind coxa nearly up to apex of third tergite (Fig. 14), mainly rather sparsely punctate but dorso-apically densely punctate and with some striae; length of hind femur, tibia and basitarsus 3.4, 5.2 and 4.0 × their width, respectively (Fig. 15); outer apical half of hind tibia and ventrally hind tarsus with dark brown spines; length of outer and inner spur of middle tibia 0.5 and 1.0 × middle basitarsus, respectively and inner spur curved (Fig. 11); length of outer and inner spur of hind tibia 0.5 and 0.7 × hind basitarsus, respectively and inner spur straight; tarsal claws without lobe.

*Metasoma*. Metasoma parallel-sided in dorsal view (Fig. 13); first tergite short, parallel-sided apically, mainly longitudinally rugulose, its median carina nearly as long as dorsal face of tergite ending in a smooth triangular area and crenulate grooves along dorsal carinae X-shaped (Fig. 13); medial area of second tergite vase-shaped, largely smooth but anteriorly superficially punctate and its surroundings coarsely longitudinally rugose (Fig. 13); third tergite coarsely irregularly rugose, but medially superficially sculptured and sublaterally depressed and medially 1.2 × longer than second tergite; ovipositor sheath 0.11 × as long as fore wing and 0.7 × hind basitarsus, narrow (Fig. 14).

*Colour*. Black; palpi, tibial spurs and tegula white; clypeus, mandible, galea, humeral plate, scapus and pedicellus (except brown stripe), third-fifth antennal articles ventrally, legs (but apical half of hind tibia and tarsus (except pale yellow basal 0.4 of hind basitarsus) dark brown), anterior half of metasoma ventrally, wing veins (but 1-M, 1-CU1 and cu-a brown) pale yellow; fore leg, middle leg (but coxa brown and femur yellowish-brown) and metasoma yellow; pterostigma (except basally) and fourth-seventh tergites dark brown; apex of hind femur, hypopygium and ovipositor sheath largely brown; wing membrane subhyaline.

*Variation.* Length of body 2.3–2.5 mm, of fore wing 2.6–2.8 mm; vein 1-R1 of fore wing 1.0–1.2 × as long as pterostigma; medial area of second tergite mainly distinctly rugose or superficially punctate. Male is very similar and has vein 1-CU1 0.6 times vein 2-CU1.

#### Distribution.

Vietnam.

#### Biology.

Unknown. Adults collected in AprilMay.

#### Etymology.

Name derived from “nigro” (Latin for “blacken”) and “soma” (Greek for “body”) because of the mainly black body.

### Pseudofornicia vanachterbergi

Taxon classificationAnimaliaHymenopteraBraconidae

Long nom. n.

Figs 21 , 22–30

Fornicia achterbergi Long, 2007: 37–38, 41–42, figs 7–15 (not *Fornicia achterbergi* Yang & Chen, 2006).

#### Type material.

Holotype, ♀ (IEBR), “VN [= Vietnam]: Hà Tây, Thach Thât, vuòn chè, M[alaise]T[rap], 25.v–5.vi.2002, K.D. Long”.

#### Diagnosis.

Head 0.9 × as wide as mesoscutum in dorsal view and height of head 0.6 × height of mesosoma in lateral view (Fig. 21); scutellum medially distinctly rugose (Fig. 28), its apical rim nearly reaching upper level of scutellum (Fig. 29); first discal cell less setose than apical third of fore wing; vein m-cu of fore wing shorter than vein 2-SR+M (Fig. 22); vein cu-a of hind wing moderately sinuate (Fig. 24); apical half of hind tibia yellowish brown (Fig. 25); metasoma brownish yellow dorsally and roundly narrowed posteriorly (Fig. 23); first tergite near median carina depressed (Fig. 23); propodeum with small elevated medio-basal area (Fig. 28); medial area of second tergite largely subparallel-sided and only anteriorly widened (Fig. 23), its anterior half largely smooth, except some punctures laterally and area rather abruptly narrowed posteriorly (Fig. 23); second and third tergites brownish yellow medially (Fig. 23); third tergite rather coarsely reticulate medially, its median length 1.3 × second tergite (Fig. 23).

#### Distribution.

Vietnam.

#### Biology.

Unknown, but reared from a host on a litchi tree.

#### Notes.

Dr Khuat Dang Long renames here his *Fornicia achterbergi* Long, 2007, into *Pseudofornicia vanachterbergi* nom. n., because it is a primary homonym of *Fornicia achterbergi* Yang & Chen, 2006.

## Supplementary Material

XML Treatment for Pseudofornicia

XML Treatment for Pseudofornicia commoni

XML Treatment for Pseudofornicia flavoabdominis

XML Treatment for Pseudofornicia nigrisoma

XML Treatment for Pseudofornicia vanachterbergi
